# Increased Leptin Levels in Plasma and Serum in Patients with Metabolic Disorders: A Systematic Review and Meta-Analysis

**DOI:** 10.3390/ijms252312668

**Published:** 2024-11-26

**Authors:** Yazmín Hernández-Díaz, María de los Ángeles Ovando-Almeida, Ana Fresán, Isela Esther Juárez-Rojop, Alma Delia Genis-Mendoza, Humberto Nicolini, Thelma Beatriz González-Castro, Carlos Alfonso Tovilla-Zárate, María Lilia López-Narváez

**Affiliations:** 1 División Académica Multidisciplinaria de Jalpa de Méndez, Universidad Juárez Autónoma de Tabasco, Jalpa de Méndez 86205, Tabasco, Mexico; yazmin.hdez.diaz@gmail.com (Y.H.-D.); angeles.ovando1219@hotmail.com (M.d.l.Á.O.-A.); 2 Subdirección de Investigaciones Clínicas, Instituto Nacional de Psiquiatría Ramón de la Fuente Muñiz, Ciudad de México 14370, Mexico; a_fresan@yahoo.com.mx; 3 División Académica de Ciencias de la Salud, Villahermosa, Universidad Juárez Autónoma de Tabasco, Villahermosa 86040, Tabasco, Mexico; iselajuarezrojop@hotmail.com; 4 Servicio de Atención Psiquiátrica, Hospital Psiquiátrico Infantil Dr. Juan N. Navarro, Ciudad de México 14080, Mexico; adgenis@inmegen.gob.mx; 5 Laboratorio de Genómica de Enfermedades Psiquiátricas y Neurodegenerativas, Instituto Nacional de Medicina Genómica, Ciudad de México 14610, Mexico; hnicolini@inmegen.gob.mx; 6 División Académica Multidisciplinaria de Comalcalco, Universidad Juárez Autónoma de Tabasco, Comalcalco 86658, Tabasco, Mexico; dralilialonar@yahoo.com.mx

**Keywords:** chronic diseases, metabolism, leptin, diabetes, obesity, meta-analysis

## Abstract

A large number of studies have reported the relationships between leptin levels and diabetes or obesity. However, the results are still controversial, and no consensus has been reached. Therefore, the purpose of the study was to collect data from various databases to perform a meta-analysis and address the inconsistencies in these studies. A systematic literature search was conducted on PubMed, Web of Science, and EBSCO for relevant available articles. The pooled standard mean difference (SMD) with 95% confidence interval (CI) was used to estimate the association by a meta-analysis. Fifteen reports with 1,388 cases and 3,536 controls were chosen for the meta-analysis. First, an increase in leptin levels in serum (SMD 0.69; 95% CI 0.36–1.02 ng/mL) and plasma (SMD 0.46; 95% CI 0.18–0.74 ng/mL) was observed in individuals with diabetes compared to controls. This increased level was also observed by gender and population. Second, statistical analysis showed that leptin levels in serum were significantly increased in individuals with obesity (SMD 1.03; 95% CI 0.72–1.34 ng/mL). This meta-analysis analyzed leptin in individuals with diabetes or obesity and emphasized the importance of monitoring serum/plasma leptin levels in patients with these diseases. However, more comprehensive studies are necessary in order to draw firm conclusions.

## 1. Introduction

Obesity and diabetes are global health problems and risk factors for premature death. Patients with obesity are more likely to develop type 2 diabetes mellitus (T2DM) which has become one of the most prevalent chronic diseases worldwide, affecting more than 250 million people [1]. Both obesity and diabetes have been related to several cardiovascular, neurological, and other clinical complications. In relation to this, the physiopathology of obesity and T2DM is frequently associated with alterations in lipids and glucose metabolism, insulin signaling, adipose tissue development and inflammation, among others [2].

Leptin is a peptide hormone secreted by adipocytes, with a crucial role in the regulation of body mass through a negative feedback mechanism between adipose tissue and the hypothalamus [3]. In this sense, leptin acts as an appetite-regulating factor that induces a decrease in food intake and an increase in energy consumption by inducing anorexigenic factors and suppressing neuropeptides [4]. Thus, alterations in leptin levels are highly associated with metabolic comorbidities such as obesity and diabetes.

Several studies have been carried out to determine the association between leptin levels and obesity or diabetes. For example, Huang et al. [5] conducted a study and observed elevated serum leptin levels in patients with T2DM. On the contrary, the data provided by Yin et al. [6] did not confirm this association, since they observed reduced leptin levels in patients with diabetes compared to healthy controls. Other studies suggest that leptin resistance is a key factor in the development of obesity. However, the leptin levels have been reported responding to changes in feeding and adiposity in the population. Therefore, leptin expression and circulating levels increase and reflect the degree of adiposity [7].

In view of that, understanding the role of leptin in metabolic disorders is indeed complex, and due to the discrepancies in the findings of previous studies that suggest that leptin levels could be a consequence of the pathologies and not a prognostic marker, we performed a meta-analysis to investigate the role of leptin in individuals with diabetes or obesity. We will also explore sources of heterogeneity between studies using subgroup meta-analysis and meta-regression.

## 2. Materials and Methods

### 2.1. Design

The systematic review and meta-analysis followed the recommendations of the Preferred Reporting Items for Systematic Reviews and Meta-Analyses (PRISMA) guidelines. The protocol of this systematic review was registered in the International Prospective Register of Systematic Reviews (PROSPERO) (protocol ID: CRD42023389891).

#### Search Strategy

**Figure 1:**
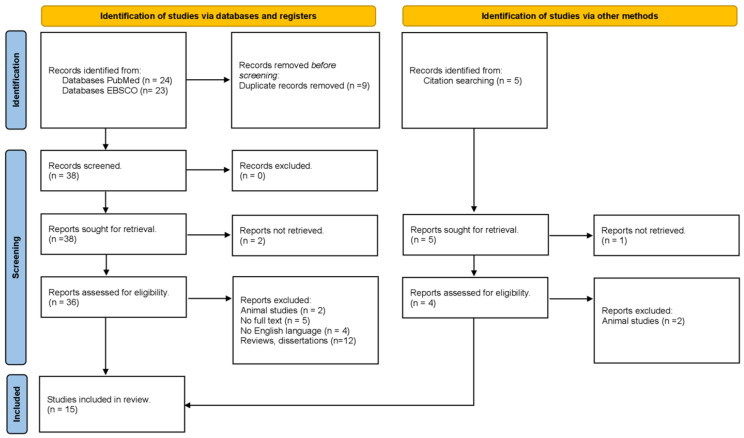
PRISMA flowchart of the inclusion process.

A systematic search of the PubMed and EBSCO databases up to February 2023 was conducted using medical subject headings (MeSH) or free-text words. The search keywords were “leptin” and “diabetes” or “obesity”. The references cited in the studies and in review articles were also examined to identify additional reports. These studies were screened by reading their titles, abstracts, and contents (Figure 1). Only published studies with full-text reports were included.

### 2.2. Inclusion/Exclusion Criteria

Articles were included when they met the following criteria: (1) Patients with clinical diagnoses of obesity or with diagnoses of diabetes; (2) A control group; (3) Leptin levels of cases and controls (mean ± SD); (4) English language; (5) Articles published in peer-reviewed journals; (6) Whole information available in the article. Articles were excluded if: (1) The data were not fully available after contacting the authors by email; (2) Reviews or meta-analyses; (3) Duplicated studies.

### 2.3. Data Extraction

All data were extracted independently by two reviewers (YHD and MAOA) according to the inclusion and exclusion criteria. The following data were extracted from the articles: first author’s name, year of publication, country, type of biological sample, diagnostic, total leptin levels, and population size of cases and controls. Disagreements were initially discussed and then resolved by a third independent author (CATZ).

### 2.4. Quality Appraisal

The Newcastle–Ottawa Scale (NOS) was used to assess study quality and adapted for cross-sectional studies. The NOS scale assesses the methodological quality of three categories of studies: study selection, group comparability, and exposure. A total of nine stars can be rated according to certain criteria: for the selection category, nine stars can be awarded, a maximum of two stars for the comparability category, and three stars for the result category. The scores obtained on the NOS scale were then used to assign study quality as good (>6 stars) or poor (<6 stars).

### 2.5. Statistical Analyses

Comprehensive Meta-analysis version 2 software (Biostat, Englewood, NJ, USA) was used to treat quantitative data. Standardized mean differences (SMD) and their 95% confidence intervals (CIs) were calculated and represented the differences in mean leptin levels between individuals with obesity or diabetes, and healthy individuals as controls. Heterogeneity was assessed using the Q test (*p* < 0.05) and the I^2^ test (>50%).

Subgroup and meta-regression analyses were performed to address the reasons for heterogeneity. The subgroup analysis was conducted based on gender, ethnicity, and biological sample. Meta-regression was performed to investigate whether covariates (sample size and mean age) accounted for the observed effect. Sensitivity analyses were conducted to assess the stability of the pooled results. Possible publication bias was explored using Egger’s linear regression test (Egger’s test). The value of *p* < 0.05 was considered statistically significant.

## 3. Results

### 3.1. Literature Screening Process and Results

Figure 1 indicates the overall flow of the study selection, literature search, and number of the included studies. At the end of the search, a total of 52 articles were obtained and filtered; for example, duplicate articles were eliminated (n = 9). Reviews and dissertations were then eliminated (n = 12). Articles were then reviewed to ensure that they covered all three aspects of the inclusion criteria (diabetic patients, obese patients, and leptin levels). After a meticulous review of each article, 15 studies met the inclusion criteria [5,6,8,9,10,11,12,13,14,15,16,17,18,19,20].

### 3.2. Methodological Characteristics of the Studies

The characteristics of the included studies are shown in Table 1. The sample size of the 15 included studies ranged from 3536 for controls and 1388 cases (people with diabetes/obesity), with a total of 4924 participants. Of the 15 studies analyzed, only two showed data from individuals with obesity and diabetes [14,19]. The selected studies were divided into groups and subgroups; for example, in the analysis, the diabetic population was grouped into diabetics, healthy subjects, according to their ethnicity, and biological sample. On the other hand, studies with obese populations were grouped into obese, healthy subjects, and according to the biological sample used in the analysis.

### 3.3. Meta-Analysis of Leptin Levels in Individuals with Diabetes vs. Controls

**Figure 2:**
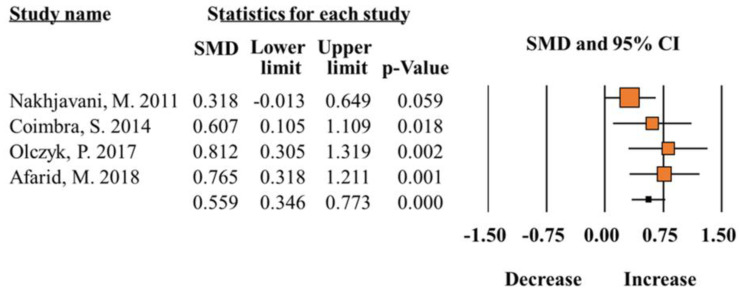
Meta-analysis of leptin levels in patients with diabetes compared to healthy controls (I^2^ = 21.72) [12,15,16,17].

A total of 12 studies [6,8,9,10,12,13,14,15,16,17,18,19] contained analyzable data and were included in the meta-analysis. The analysis indicated that total leptin in blood was significantly increased in individuals with diabetes, compared to controls (SMD 0.55; 95% CI 0.34–0.77; *p* < 0.0001; Figure 2) (Table 2). According to subgroup results, increased SMD of total leptin levels was significantly associated with sex (Male, SMD 0.34; 95% CI 0.17–0.50; *p* < 0.0001; Female, SMD 0.34; 95% CI 0.16–0.53; *p* = 0.00) and biological sample (Plasma, SMD 0.46; 95% CI 0.18–0.74; *p* < 0.0001; Serum, SMD 0.69; 95% CI 0.36–1.02; *p* = 0.00). Regarding the ethnicity, diabetic patients (Middle East) showed increased leptin levels compared to controls (SMD 0.47; 95% CI 0.21–0.74; *p* < 0.0001). However, the results were inversely associated with SMD difference of leptin (SMD −0.76; 95% CI −0.95–−0.57; *p* < 0.0001) in the Caucasian population. The heterogeneity test indicated that homogeneity was ideal for all analysis (I^2^ = < 50%, *p* > 0.05) (Table 2).

### 3.4. Meta-Analysis of Total Leptin Levels in Individual with Obesity vs. Controls

**Figure 3:**
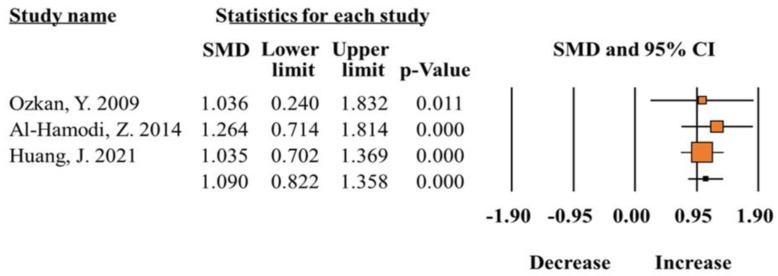
Meta-analysis of leptin levels in patients with obesity compared to healthy controls (I^2^ = 00.00) [5,11,14].

A total of five studies [5,11,12,19,20] reported the comparison of total leptin levels of obese patients and controls. The results showed that the difference between total leptin levels of obese patients compared with controls was statistically significant (SMD 1.09; 95% CI 0.82–1.35; *p* = 0.00; Figure 3) (Table 2). The increase in total leptin levels was further confirmed with serum concentrations between obese patients and controls (SMD 1.03; 95% CI 0.72–1.34; *p* = 0.00). The heterogeneity test showed good homogeneity (I^2^ =< 50%, *p* > 0.05).

### 3.5. Evaluation of Publication Bias

**Figure 4:**
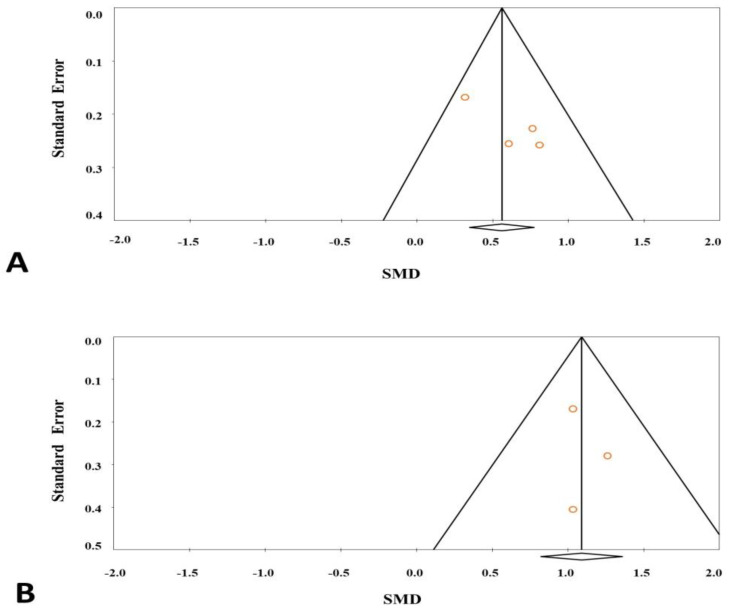
Funnel plot for studies in leptin levels for subjects with diabetes (**A**) or obesity (**B**) versus control subjects.

Publication bias was analyzed by using the Begg’s test and Egger’s test. The results showed that there was no publication bias in the results (Figure 4). The statistical results of publication bias are shown in Table 2.

### 3.6. Meta-Regression and Sensitivity Analysis

Meta-regression analysis was conducted to examine the effect of the covariates on effect size. The covariates, namely sample size (β = 0.40, *p* = 0.64) and mean age (β = 0.85, *p* = 0.77), did not have any effect on leptin levels. Sensitivity analysis was performed to examine the influence set by the individual study on the pooled SMD, by sequentially excluding each case-control study. Consistently, our data were stable and reliable in all meta-analyses.

### 3.7. Quality Appraisal

Our quality assessment using the NOS scale for studies is presented in Table 3. The mean total score of NOS was 7.1. Therefore, all studies were identified as of good methodological quality due to the low risk of bias (scores > 6).

## 4. Discussion

Metabolic syndrome comprises a cluster of cardiometabolic risk factors that include obesity, hyperglycemia, hypertension, and dyslipidemias. The growing prevalence of metabolic syndrome is becoming a serious health problem and economic burden; moreover, the difficulty in the management of metabolic syndrome is linked to its multifactorial nature. Obesity and diabetes are strongly associated with the prognosis of other diseases such as atherosclerosis. Adipose tissue secretes adipokines that affect whole-body metabolism. Evidence suggests that adipokines such as adiponectin, leptin, and interleukin-6 can play important roles in atherosclerosis development, progression, as well as regression [7,21]. Therefore, the monitoring of blood adipokines levels in patients with these diseases is important. Here, we explore the correlation between leptin levels and diabetes or obesity through a meta-analysis. Our findings demonstrate that increased leptin levels are associated with increased risk of diabetes or obesity, compared with population control subjects.

Leptin plays an important role in the pathophysiology of metabolic syndrome. Leptin is primarily produced by white adipose tissue; it decreases insulin sensitivity and, therefore, could lead to decreased glucose tolerance. The insulin-producing β cell may be negatively affected by chronically elevated leptin levels, leading to decreased responsiveness of the receptor system in β cells, resulting in failure to suppress insulin secretion. The resulting hyperinsulinemia could, in turn, exacerbate obesity and further increase leptin levels and gene expression in white adipose tissue. Therefore, elevated leptin levels may contribute to obesity and insulin resistance as a consequence of this, in a positive feedback loop, may promote the development of metabolic disorders [22]. Kim et al. demonstrated that obesity and the chronic consumption of high-fat diets produce significant changes in the blood–brain barrier (BBB) [23], and also in brain regions that contain neurons with high metabolic demands, such as those of the arcuate core of the hypothalamus and hippocampus. The arcuate nucleus (ARH) is an important leptin-sensing site because it converts peripheral signals into neuronal responses [24].

Additionally, in the present study, when leptin levels were compared in individuals with obesity, they showed higher leptin levels (point estimate 1.09, lower-upper range: 0.82–1.35) than individuals with diabetes (point estimate 0.55, lower-upper intervals: 0.34–0.77). Although plasma leptin levels are high, impaired leptin transport to the brain among obese individuals results in leptin resistance [25]. In addition to the above, it is known that the main cause of low leptin levels is related to the deficiency of its transport through the blood–brain barrier; that is, the leptin transport mechanism is saturated or defective in people with obesity [26]. In fact, this hormone plays an important role in the negative feedback loop between adipose tissue and the brain. Therefore, the combination of high levels of leptin and serum triglycerides may be a marker of obesity “at risk” [27]. Adipose tissue dysfunction in obesity causes hypertriglyceridemia; this is due to increased hepatic production of very-low-density lipoproteins (VLDL), as well as decreased triglyceride hydrolysis [28]. Consequently, triglycerides inhibit the transport of leptin through the blood–brain barrier, so that its levels rise in the brain and redirect the use of calories toward food-seeking activities.

Racial and ethnic differences in obesity and diabetes require a thorough understanding of mediator factors, including lipid, hormone, and lipoprotein metabolism. Lipid levels are positively associated in people with metabolic disorders; for example, Pan et al. [29] and Colin et al. [30] specifically compared obesity-related risk factors. Their results suggest that increases in BMI correspond to higher odds ratios (ORs) in Chinese compared with Caucasians for hypercholesterolemia, hypertriglyceridemia, and diabetes. In fact, Gaillard et al. [31] confirmed that serum triglycerides, measured by traditional enzymatic methods, are significantly lower in African-American women than in American women with prediabetes. The above findings are consistent with our report and those of other investigators because we observed a notable difference in the analysis by populations. For example, in studies with the Caucasian population, it was observed that this significant association is related to a decrease in levels of leptin. Therefore, we can conclude that lipids play a fundamental role in metabolic disorders. This trend among the aforementioned populations can be attributed to a strong genetic–environmental interaction, as it has been intensified by rapid lifestyle changes in a growing economy.

Although there have been several studies on the relationship between leptin levels and diabetes or obesity, the conclusions have not been consistent. In recent years, attention has focused on the role of visceral adipose tissue due to the synthesis and release of a number of adipokines (leptin and adiponectin) from adipocytes. There are a number of physiological and metabolic changes associated with obesity that may contribute to increased leptin levels [7]. Due to the above, our results should be taken with caution, since the positive association observed could be a consequence of the pathologies, and not a prognostic marker. It will be necessary to verify the results and clarify the mechanism through which this may occur.

Some limitations of the present meta-analysis need to be addressed. First, although the present analysis includes 15 studies, it is relatively small compared to other meta-analyses on such conditions. We consider that the number of eligible studies included in our meta-analysis is small, so to validate our results we suggest including a larger number of studies in future research. On the other hand, the analysis of subgroups by ethnicity revealed an important association in Caucasian populations; however, there are few studies based on this population, so we could not make a comparison to see if there is any change in leptin levels in people with obesity or diabetes related to ethnicity. We suggest further studies investigating this association in Caucasian and other populations.

## 5. Conclusions

In conclusion, this current meta-analysis demonstrated that leptin levels were significantly upregulated in cases, compared with those in the controls. However, we suggest conducting further studies with a larger number of patients and in diverse populations to verify the results and clarify the mechanisms involved.

## Figures and Tables

**Table 1:** Main characteristics of eligible studies.

Author	Country	Year	Population					Main Results	
			Sample	Ethnicity	Total	Control	Case	Study	
Hanaki et al. [8]	USA	1999	Serum	Caucasian	38	19	19	Diabetes	The results of this study demonstrate that patients with new-onset type 1 diabetes have low leptin levels.
Tatti et al. [9]	Italy	2001	Plasma	Caucasian	300	160	140	Diabetes	The leptin levels were lower in the diabetic population only when both sexes were combined, and were higher in the females of both groups.
Al-Daghri et al. [10]	Asia	2007	Serum	Afro-American	222	80	142	Diabetes	Leptin is associated with measures of adiposity (hip circumference, in particular) in the non-diabetic state among Saudi subjects.
Ozkan et al. [11]	Turkey	2009	Serum	Caucasian	31	10	21	Obesity	Leptin levels were lower in controls than in obese subjects.
Nakhjavani et al. [12]	Iran	2011	Plasma	Middle East	142	71	71	Diabetes	Leptin concentration correlated with obesity in female, but not male, diabetic subjects.
Morteza et al. [13]	Iran	2013	Serum	Middle East	85	41	44	Diabetes	A positive correlation between leptin levels in patients with T2DM was reported.
Al-Hamodi et al. [14]	Republic of Yemen	2014	Plasma	Caucasian	92	46	46	Diabetes and obesity	Leptin levels were higher in both obese subjects and non-obese T2DM patients.
Coimbra et al. [15]	Portugal	2014	Serum	Portuguese	93	20	73	Diabetes	Leptin levels in elderly patients with T2DM seem to be closely linked to obesity and to length of the disease.
Olczyk et al. [16]	Poland	2017	Plasma	Yemeni	67	27	40	Diabetes	Leptin values were significantly higher in patients with untreated T2DM.
Yin et al. [6]	USA	2018	Plasma	Caucasian	124	63	61	Diabetes	This study showed that a high level of leptin is associated with improved cognitive function in T2DM patients and more significantly in female individuals.
Afarid et al. [17]	Iran	2018	Serum	Middle East	83	39	44	Diabetes	Increased serum levels of leptin were associated with advanced stages of diabetic retinopathy in subjects with T2DM.
Bidulescu et al. [18]	USA	2020	Plasma	Caucasian	3363	2779	584	Diabetes	The association of leptin with incident T2DM was mediated by insulin resistance.
Katsogiannos et al. [19]	Sweden	2021	Plasma	Swedish	59	25	34	Diabetes and obesity	Leptin values were higher in all patients with obesity compared with healthy controls.
Sitar-Taut et al. [20]	Romania	2021	Serum	Caucasian	29	12	17	Obesity	Leptin levels were significantly higher in obese and diabetic patients.
Huang et al. [5]	China	2021	Serum	Asian	196	144	52	Obesity	Leptin values were higher for diabetic nephropathy in the T2DM patients.

T2DM, Type 2 Diabetes Mellitus.

**Table 2:** Analysis of leptin levels in patients with diabetes or with obesity.

Subgroup Analysis	Pooled Data				Heterogeneity		Egger *p*-Value
	Point Estimate	Lower	Upper	Z *p*-Value	Q *p*-Value	I^2^	
Diabetic vs. Healthy	0.559	0.346	0.773	0.000	0.280	21.727	0.10774
Subgroup							
Male	0.340	0.172	0.509	0.000	0.225	31.236	0.63029
Female	0.348	0.160	0.537	0.000	0.311	14.378	0.56557
Middle East	0.477	0.211	0.743	0.000	0.0116	59.583	0.39321
Caucasian	−0.763	−0.953	−0.574	0.000	1.185	0.553	0.33844
Plasma	0.466	0.189	0.743	0.001	0.110	60.788	0.65302
Serum	0.695	0.361	1.029	0.000	0.646	0.000	0.71815
Obese vs. Healthy	1.090	0.822	1.358	0.000	0.777	0.000	0.73765
Subgroup							
Serum	1.036	0.728	1.343	0.000	1.000	0.000	1.000

**Table 3:** Quality assessment of included studies according to the modified Newcastle–Ottawa Scale (NOS).

Author	NOS Category			Assessment
	Selection	Comparability	Outcome	
Hanaki K. [8]	⋆⋆	⋆⋆	⋆⋆	Good
Tatti P. [9]	⋆⋆⋆	⋆⋆	⋆⋆	Good
Al-Daghri N.M. [10]	⋆⋆⋆⋆	⋆⋆	⋆⋆	Good
Ozkan Y. [11]	⋆⋆⋆	⋆⋆	⋆⋆	Good
Nakhjavani M. [12]	⋆⋆⋆⋆	⋆⋆	⋆⋆	Good
Morteza A. [13]	⋆⋆⋆	⋆⋆	⋆⋆	Good
Al-Hamodi Z. [14]	⋆⋆⋆⋆	⋆⋆	⋆⋆	Good
Coimbra S. [15]	⋆⋆	⋆⋆	⋆⋆	Good
Olczyk P. [16]	⋆⋆	⋆⋆	⋆⋆	Good
Yin H. [6]	⋆⋆⋆⋆	⋆⋆	⋆⋆	Good
Afarid M. [17]	⋆⋆⋆	⋆⋆	⋆⋆	Good
Bidulescu A. [18]	⋆⋆⋆	⋆⋆	⋆⋆	Good
Katsogiannos P. [19]	⋆⋆⋆	⋆⋆	⋆⋆	Good
Sitar-Taut A. V. [20]	⋆⋆⋆⋆	⋆⋆	⋆⋆	Good
Huang J. [5]	⋆⋆⋆	⋆⋆	⋆⋆	Good

The star represents a point.
